# Skeletal Muscle Mitochondria Dysfunction in Genetic Neuromuscular Disorders with Cardiac Phenotype

**DOI:** 10.3390/ijms22147349

**Published:** 2021-07-08

**Authors:** Elena Ignatieva, Natalia Smolina, Anna Kostareva, Renata Dmitrieva

**Affiliations:** 1 Almazov National Medical Research Centre, 197341 St. Petersburg, Russia; smolina_na@almazovcentre.ru (N.S.); anna.kostareva@ki.se (A.K.); 2 Department of Woman and Child Health, Karolinska Institute, 17177 Stockholm, Sweden

**Keywords:** neuromuscular disorders, cardiomyopathies, mitochondrial dysfunction

## Abstract

Mitochondrial dysfunction is considered the major contributor to skeletal muscle wasting in different conditions. Genetically determined neuromuscular disorders occur as a result of mutations in the structural proteins of striated muscle cells and therefore are often combined with cardiac phenotype, which most often manifests as a cardiomyopathy. The specific roles played by mitochondria and mitochondrial energetic metabolism in skeletal muscle under muscle-wasting conditions in cardiomyopathies have not yet been investigated in detail, and this aspect of genetic muscle diseases remains poorly characterized. This review will highlight dysregulation of mitochondrial representation and bioenergetics in specific skeletal muscle disorders caused by mutations that disrupt the structural and functional integrity of muscle cells.

## 1. Introduction

Inherited cardiomyopathies (CM) are a group of monogenic cardiovascular disorders characterized by ventricular dysfunction and heart failure, and the primary structural and functional defects arise in the cardiomyocytes [1]. Twenty-five percent of the known cardiomyopathy-related genes also affect skeletal muscle as allelic forms causing neuromuscular disorders [2,3]. Inherited neuromuscular disorders clinically are often combined with cardiac phenotype presenting in the form of cardiomyopathies or arrhythmic disorders due to the expression of mutated genes in both skeletal muscle cells and cardiomyocytes [4]. Most of these genes encode the structural proteins of striated muscle cells. Therefore, the deficiency of muscle structural proteins results in cardiac and skeletal forms of myopathies and myodystrophies, characterized by progressive wasting, weakness, and damage to muscle tissue.

Skeletal muscle is one of the most metabolically active tissue types with particularly high energetic demands, making it very susceptible to defects in mitochondrial function. It is becoming increasingly clear that alterations in mitochondrial capacity and quality play a central pathophysiological role in the loss of function associated with different muscle-wasting conditions (for review see [5,6]), including heart failure [7], Duchenne muscular dystrophy [8], and sarcopenia [9]. A broad spectrum of mitochondrial derangements occurs in skeletal muscle cells resulting in the pathogenesis of the neuromuscular disorders: alterations in morphology and dynamics, impaired energy production, enhanced ROS (reactive oxygen species) generation, and dysregulated biogenesis [6].

However, the precise mechanisms that link mitochondrial dysfunction and the development of muscle wasting conditions in CM have not yet been described. Moreover, this aspect of the inherited muscular disorders remains poorly characterized. This review aims to present the current state of knowledge regarding skeletal muscle mitochondrial bioenergetics and metabolism in genetically determined neuromuscular diseases associated with cardiomyopathies. Here, we summarize the available data supporting mitochondrial dysfunction as a key event in the development of skeletal myopathies due to mutations that violate the structural and functional integrity of the muscle cell.

## 2. Role of Mitochondria in Muscle

Both the heart and skeletal muscle tissue types are highly dependent on the energy supply needed to produce contractions. Together, they account for nearly 30% of resting energy consumption and almost 100% of the increase in energy consumption during exercise [10]. Energy metabolism in these tissues is tightly regulated to meet the energy needs and might be pathologically affected in patients with cardio- and skeletal myopathies.

The energy in skeletal muscle is derived from glucose and fatty acids, supplied by blood and stored in the muscle fibers in the form of glycogen and intramyocellular lipids (mainly in the form of triglycerides). The main fuel in muscle is the energy-rich phosphate compound adenosine 5′-triphosphate (ATP), which is mostly produced by the oxidative phosphorylation system (OxPhos) composed of five (I-IV and complex V, ATP synthase) multi-oligomeric complexes, as well as the electron transporters ubiquinone and cytochrome C, of the electron transport chain (ETC) in the inner mitochondrial membranes. Moreover, mitochondria serve as an important source of reactive oxygen species (ROS), vital second messengers; however, ROS overproduction might lead to oxidative stress. Mitochondria are also involved in the maintenance of lipid homeostasis and regulation of apoptotic cell death via mitochondrial permeability transition pore (MPTP) formation [11].

Mitochondria are strategically distributed in cells, and their bioenergetic function is highly dependent on ultrastructure and morphology [12,13]. Thus, alterations in mitochondrial content, positioning, morphology, and physiology may result in altered ability to adapt rapidly bioenergetic machinery to meet energy demands, which in turn results in a decrease in quality of life and worsening of prognosis in patients with neuromuscular disorders and cardiomyopathies.

Even before the genetic background of cardiomyopathies was proved, it was shown that skeletal muscle abnormalities occur in patients with cardiomyopathies: pathological structural, functional, and metabolic changes in skeletal muscle of patients were demonstrated by histological, electromyographic, and magnetic resonance imaging evaluations [14,15,16,17,18,19,20,21]. Several early studies have identified mitochondrial abnormalities in skeletal muscle of CM patients: subsarcolemmal mitochondrial proliferation and ultrastructural alterations [15]; swelling with clarification of the matrix and cristolysis [16,22]; reduced mitochondrial enzymatic activity [16]; mitochondrial hyperplasia [23]; focal reduction in mitochondria in the center of type I oxidative fibers [24]; and mitochondrial DNA (mtDNA) mutations [22].

## 3. Mitochondrial Dysfunction in Specific Neuromuscular Disorders

### 3.1. Dystrophinopathies

Dystrophin is a part of a dystrophin-glycoprotein complex (DGC) in cardiac and skeletal muscle cells, which provides a mechanical link between the cytoskeleton and the extracellular matrix and is responsible for the stabilization of the plasma membrane of striated muscle cells, especially during muscle contraction [25,26]. Besides, DGC is involved in the regulation of signal transduction, thus mediating interactions between the cytoskeleton, membrane, and the surrounding extracellular matrix [27,28,29,30]. Loss of normal dystrophin function leads to both Duchenne and Becker forms of muscular dystrophy (DMD and BMD), a progressive muscle-wasting disease in boys, as well as to X-linked dilated cardiomyopathy. In DMD, dystrophin is almost absent; whereas, in BMD, it decreases in size or amount. Cardiovascular complications occur in >80% of dystrophin-associated myodystrophies, and most patients with DMD and BMD have severe cardiac dysfunction by the third decade of life [31,32]. There are many excellent reviews regarding dystrophinopathies that describe the clinical picture in detail [2,33,34,35,36].

The molecular pathophysiology of DMD has been attributed to dysregulation of Ca^2+^ handling, resulting in elevated levels of intracellular Ca^2+^ [37,38], which contribute to calpain activation followed by degradation of muscle proteins [39,40]. Unbalanced Ca^2+^ cycling could be caused by the enhanced entry from the extracellular space or by non-sufficient uptake by an intracellular depot [30]. In skeletal muscle, intracellular release and uptake of Ca^2+^ are mainly controlled by the sarcoplasmic reticulum (SR), which is physically interconnected and functionally linked to mitochondria, allowing the direct transfer of calcium from SR to these organelles. In *mdx* mice, the most commonly used mouse model of DMD, an impaired SR-mitochondria interaction was shown to be associated with derangement in Ca^2+^ handling by mitochondria [41,42]. Studies of DMD molecular pathogenesis performed in vitro and in vivo demonstrated boosted activity of purinergic receptors [43], over-expression, and upregulation of the sarcolemmal Ca^2+^ channels, e.g., mechanosensitive transient receptor potential channels [44,45,46] and participants of store-operated calcium entry pathway. The intrinsic machinery responsible for the elevated intracellular Ca^2+^ levels involves leaky ryanodine receptors in the sarcoplasmic reticulum [47,48] and also declined ability of mitochondria to buffer Ca^2+^ overload and to provide ATP needed for repairing the sarcolemmal injury [49].

In several recent works, a group of authors provided evidence for a significant dysregulation of mitochondrial calcium homeostasis in skeletal muscle of *mdx* mice: a decreased rate of calcium influx and lower Ca^2+^ capacity of DMD muscle mitochondria, compared with wild type organelles. These abnormalities may be due to the altered expression of components of the systems of specific calcium transport and MPTP opening, both at the mRNA and protein level. Thus, a lower rate of Ca^2+^ uptake was accompanied by an increased content of MCUb, an inactive channel subunit of calcium uniporter in the inner mitochondrial membrane. The observed decline in Ca^2+^ capacity of *mdx* mitochondria (the maximal amount of calcium ions in the matrix that does not cause MPTP opening) correlated with their higher sensitivity to the Ca^2+^ dependent induction of MPTP. This may be associated with an elevated level of ANT2 (adenine nucleotide translocase 2), and reduced levels of ANT1, cyclophilin D, α-subunit of mitochondrial ATP synthase, and VDAC1, which are the putative components of the pore apparatus [50]. Treatment with glucocorticoid deflazacort had beneficial effects on muscle function through normalization of mitochondrial respiration and calcium homeostasis possibly due to rearrangements in the mitochondrial proteome: an increase in the level of some ETC complexes, including ATP synthase, and a decrease in the level of MCUb [51]. At the same time, deflazacort reduced the resistance of *mdx* skeletal muscle mitochondria to MPTP opening, additionally causing an increase in the ANT2 protein level. Interestingly, heart mitochondria of young *mdx* mice demonstrated, on the contrary, an increased rate of Ca^2+^ uptake, a decreased expression of MCUb subunit, and a higher resistance to MPTP opening, with a nonchanged level of MPTP putative proteins [52]. Thus, cellular pathophysiological mechanisms leading to the adaptation of organs to DMD conditions may be different in cardiac and skeletal muscle.

Mitochondrial dysfunction appears early in DMD pathogenesis and is presented by a decreased rate of mitochondrial oxidative phosphorylation and enhanced ROS generation [53,54]. The long-term effect of the cytosolic Ca^2+^ overload could be the initiation of a “vicious cycle” of mitochondrial Ca^2+^ overload and ATP depletion that leads to mitochondrial dysfunction and eventually to mitochondria-mediated cell death [55,56].

To date, metabolic and mitochondrial impairments that accompany DMD are well documented in both patients and animal models (for review, see [8,57]). The pathological features of DMD muscle include the abnormal intrafibrillar lipid accumulation [58], altered architecture, morphology, and spatial distribution of mitochondria, loss of mitochondrial biomass [59,60], disorganized mitochondrial network [53], impaired spatial control of mitochondrial localization [61], and aberrant mitochondrial morphology, namely a swollen morphological phenotype, large empty spaces inside, reduced cristae number, and abnormal cristae structure [62,63,64,65]. Alterations in mitochondrial morphology in both skeletal muscle of DMD patients and animal models are associated with abnormalities of the bioenergetic function of mitochondria, and the latter is constantly reported in the literature. These include the impaired enzymatic activity of dystrophic mitochondria (in particular, insufficiency of ETC complexes, including ATP synthase), their reduced ability for OxPhos, and, as a consequence, a reduction in ATP synthesis to 50% of healthy control levels [53,61,64,65,66,67,68,69,70,71]. In studies on the *mdx* mouse model, mitochondria isolated from muscle fibers and mitochondria in vivo demonstrated a significant reduction in basal and maximal rate of ATP synthesis when compared with the wild type control [61,68,72]. In addition, *mdx* mitochondria exhibited a decrease in the coupling of oxidative phosphorylation and electron transport chain activity [61,73]. Schuh et al. detected a 62% deficiency of the mitochondrial spare respiratory capacity in the fibers isolated from the *mdx* mice compared with the wild type mice, which means a significant decrease in the ability of these mitochondria to upregulate oxygen consumption rate in response to a growing demand for ATP [74]. Several studies have now shown that restoration of bioenergetic function of mitochondria is beneficial for muscle bioenergetics and function in the mouse model of DMD [75,76].

Interestingly, mitochondrial dysfunction is among the earliest pathological phenomena that precede the appearance of muscle damage, thus suggesting the causal role of mitochondria in the aetiology of DMD [49,65]. Since mitochondria-mediated cell death is considered an important contributor to DMD progression, there are ongoing efforts to prevent the MPTP opening and to improve the pathology in preclinical animal models. A product of fungal metabolism, a cyclic polypeptide immunosuppressant cyclosporin A (CsA) blocks MPTP through interaction and inhibition of matrix enzyme mediating MPTP opening cyclophilin D. Non-immunosuppressive CsA analogs retaining the MPTP-desensitizing properties represent a beneficial alternative to CsA. For example, *mdx* mice treated with the non-immunosuppressive derivative of CsA Debio 025 (Alisporivir) showed normal MPTP opening, enhanced mitochondrial resistance to Ca^2+^ overload, and decreased muscle necrosis [77,78]. Furthermore, the zebrafish DMD model (*sapje*), characterized by a disruption of muscle structure, a strong decrease in respiratory reserve capacity, and mitochondrial dysfunction due to the MPTP opening, showed a striking recovery after alisporivir treatment [79,80]. NIM811—another non-immunosuppressive derivative of CsA—was tested, in vivo, using a zebrafish model of collagen VI myopathy and, in vitro, using cell cultures obtained from Ullrich congenital muscular dystrophy patients. The obtained results showed the effectiveness of NIM811 in the restoration of the muscle structure and recovery of mitochondrial function [56]. The further exploration of potent and selective cyclophilin D-independent MPTP inhibitors performed by screening the small-molecule libraries identified an isoxazole compound that possessed the inhibitory activity for MPTP [81]. Further optimization work was focused on improving the pharmacokinetic properties of the identified compound, resulting in a second-generation MPTP inhibitor with improved plasma stability—TR001 [82]. Animal studies using the zebrafish model of DMD and myoblasts and myotubes from DMD patients proved the efficiency of TR001 in the restoration of mitochondrial dysfunction regulated by MPTP [83].

Besides, numerous other defects of metabolism and the cellular energy system are known to be associated with dystrophin-deficient skeletal muscle (Table S1, see Supplementary Materials).

### 3.2. Desminopathies and Myofibrillar Myopathies

Desmin is a major intermediate filament (IF) protein in both striated and smooth muscle tissue, which forms a three-dimensional framework that connects and anchors cell structures and organelles, such as desmosomes, costameres, mitochondria, and nucleus, through Z-discs, to the contractile apparatus [84,85]. It belongs to class III of IF and is encoded by a single gene (*DES*) in which multiple mutations (>70) have been identified [86] that might cause skeletal and/or cardiac myopathies. Depending on the mutation, there can be different types of familial cardiomyopathies, along with their variable combinations. It is worth noting that there is no clear correlation between the position of the *DES* mutation and concomitant clinical manifestations affecting cardiac and/or skeletal muscles to varying degrees [2,86,87].

Desmin filaments contribute to the localization and spatial distribution of mitochondria in muscle cells (reviewed in [88]), and mitochondrial dysfunction has been considered to be a key point in desmin-related pathologies, as confirmed in patients, animal, and cell models (Table S1). The most obvious evidence of the interrelation between desmin cytoskeleton and mitochondria came from studies on knockout desmin-null (*DES*^-/-^) mice. The prominent mitochondrial defects in both cardiac and skeletal muscle in desmin-null mice were discovered by Milner and co-workers [89,90]. They showed that targeted ablation of desmin led to alterations in the mitochondria positioning and morphology, which were accompanied by an impaired mitochondrial respiratory capacity, in particular, a decrease in maximum respiration in striated muscle. It is worth noting that perturbations in mitochondrial number and distribution arise before any other structural defects in muscle become obvious. Later on, it was shown that, in desmin-null mice, the soleus muscle, which is known to consist mainly of highly oxidative slow-twitch fibers with high mitochondrial content, demonstrates an abnormal accumulation of mitochondria beneath the sarcolemma [89,91,92], and have swollen and rounded mitochondria with distorted inner membranes [89,92].

Structural and functional impairments of mitochondria associated with heterozygous desmin mutations were further confirmed in the skeletal muscle of the patients [93,94]. It has been shown that complex I activity is inhibited by the expression of the mutant desmin in vivo [95,96,97]. Vincent et al. observed, in skeletal muscle from patients with desmin mutations, a decrease in mitochondrial mass associated with redistribution of mitochondria from the centre to the subsarcolemmal zone in many fibers and with deficiency of respiratory complexes I and IV [98]. In addition, in the primary myoblasts isolated from a muscle biopsy of a patient with *DES* mutation, the levels of proteins associated with the regulation of the mitochondrial permeability transition pore complex were pathologically affected [99]. Furthermore, desminopathy may even resemble mitochondrial disease, as atrophic skeletal muscles of patients were found to show significant reductions in mitochondria and mitochondrial DNA content [96]. The findings of a recent study by Kubánek et al. are in line with all data discussed above, and demonstrate variable mitochondrial dysfunctions manifested in a decreased expression of mitochondrial respiratory chain components and other mitochondrial proteins, and reduced enzyme activities in skeletal muscle of patients with *DES* mutations [100].

In a recent work, Winter and co-authors have analyzed mitochondrial function simultaneously in both muscle biopsy specimens from patients with the most frequently occurring human desmin missense mutation R350P and muscles of hetero-and homozygous knock-in mice carrying orthologous R349P mutation. In both cases, authors described violations in intracellular distribution, number, and configuration of mitochondria, as well as a decreased amount of assembled OxPhos complexes I, III, V, and other mitochondrial proteins [97]. Ultrastructural changes such as swelling, vacuolization, and increased mitochondrial Ca^2+^ level were also confirmed, in the in vivo model, in skeletal myocytes from transgenic mice harboring L345P desmin point mutation [101].

In another work using in vitro cellular models, Schröder and co-authors proved through transfection experiments that the mitochondrial abnormalities observed in a patient were the direct consequence of the expression of mutant desmin [95]. Using the lentiviral expression of six pathogenic desmin mutations, a recent work on murine primary myotubes demonstrated an affected morphology of the mitochondrial network and enhanced mtDNA release. Furthermore, the presence of *DES* mutations impaired the energetic state of skeletal muscle cells: reduced mitochondrial membrane potential, suppressed parameters of mitochondrial respiration, and increased ADP/ATP ratio, which means that the mitochondria were unable to convert effectively ADP molecules to ATP. In the other work, aggregate-prone desmin mutations were shown to suppress mitochondrial calcium uptake, presumably via the loss of desmin-mitochondria interactions due to aggregate accumulation [102,103].

Thus, accumulating evidence suggest that desmin supports the bioenergetic function of mitochondria in skeletal muscle cells. As believed, the desmin network could keep mitochondria in closeness to myofibrils, promoting the transmission of energy needs. The absence or aberrant aggregation of desmin perturbs mitochondrial network integrity and distribution, leading to mitochondrial dysfunction and abnormal energy metabolism.

It is important to mention that desminopathies belong to the group of myofibrillar myopathies (MFM), the disorders caused by the mutations in genes encoding sarcomeric and extra-sarcomeric proteins. These are, for example, ±B-crystalline (*CRYAB*), heat shock chaperone protein involved in desmin filament stabilization, and proteins of Z-disc: Cypher/ZASP (*LDB3*), filamin C (*FLNC*), myotilin (*MYOT*). As in the case of desminopathies, other MFMs are also characterized by abnormal aggregation of mutant proteins, as well as by myofibrillar destruction. Patients with MFM exhibit a cardiac phenotype as CM of different types [2]. According to a few studies, mitochondrial dysfunction is seen in the skeletal muscle of MFM patients. It manifests itself in an altered mitochondrial morphology and positioning [104]; deficiency of respiratory chain complexes I and IV (in the presence of *ZASP* and *MYOT* mutations); decreased mitochondrial mass in a high proportion of myofibers, presumably due to reduced mitochondrial biogenesis. Examination of these skeletal muscle samples also revealed a small number of clonally-expanded mtDNA deletions, indicating that the integrity of the mitochondrial network is necessary for mtDNA stability [98].

### 3.3. Lamin A and Laminopathies

Along with desmin, another important IF protein, the nuclear IF protein lamin A/C has been linked to CM, which may or may not be accompanied by neuromuscular symptoms. Lamins are the structural proteins of the nuclear lamina, which form a meshwork on the cytoplasmic side of the inner nuclear membrane providing mechanical support to the cell nucleus and connecting it with the cytoskeleton. Due to their architectural position, lamins were involved in a wide range of cellular processes including regulation of both gene expression and the activity of signaling mediators [105].

Human *LMNA* encoding A- and C-type lamins is among the most mutated genes. More than 400 distinct mutations in the *LMNA* gene are associated with over 17 different disease phenotypes known as laminopathies. Mostly, but not exclusively, these diseases affect the striated muscle, and the molecular mechanisms leading to tissue-specific phenotypes are still puzzling. Pathological phenotypes with the combined heart and skeletal muscle being affected include Emery–Dreifuss muscular dystrophy (EDMD), limb-girdle muscular dystrophy type 1B (LGMD1B), and congenital muscular dystrophy [106].

Several lines of evidence link lamins to the regulation of cell energy metabolism. When skin fibroblasts from *LMNA* mutation carriers with cardiac or skeletal muscle disorders were subjected to proteomic analysis, significant changes were found in the expression of glycolytic enzymes [107]. Signs of mitochondrial dysfunction and oxidative damage were also detected in human fibroblasts expressing lamin A mutations associated with insulin resistance and/or lipodystrophy [108], and in human fibroblasts in which the silencing of *LMNA* expression was achieved with specific siRNA [109].

Although about 80% of total *LMNA* mutations specifically affect the striated muscles [110], only one systematical evaluation of skeletal muscle metabolism in these disorders has been performed, to date. The latter was performed in a study by Boschman and co-workers [111], who observed markedly increased but incomplete skeletal muscle fatty acid oxidation, in combination with reduced glucose oxidation in patients with *LMNA*-related familial partial lipodystrophy (FPLD) and LGMD1B, in vivo and in vitro, in cultured myotubes derived from patients. In addition, microarray analysis showed down-regulation of genes involved in complex I of the respiratory chain and some glycolysis genes in FPLD myotubes.

Recently, our group, using functional and transcriptomic analysis, have discovered distinct bioenergetic changes in C2C12 myogenic cells harboring lentiviral G232E and R482L LMNA mutations, linked to different types of laminopathies associated with EDMD and FPLD, respectively [112]. Both mutations had an uncoupling effect on mitochondrial respiration in differentiated myotubes and caused an increase in proton leak, which indicate a decreased efficiency of oxidative phosphorylation. Furthermore, a reduction in myotubes’ glycolytic activity was observed in both mutant lines. In this work, we have shown that these two mutations reprogrammed muscle mitochondrial bioenergetic machinery in different directions: while in LMNA-G232E transgene myotubes we observed the substantial induction in oxidative phosphorylation, LMNA-R482L myotubes showed the suppressed mitochondrial bioenergetics.

Obviously, detailed mutation-specific bioenergetic profiles in skeletal muscle in *LMNA*-related disorders deserve further investigations.

### 3.4. Myotonic Dystrophy

Myotonic dystrophy (DM) is the most prevalent type of adult-onset muscular dystrophy. Two clinically similar types of DM, characterized by multisystem involvement with progressive muscular weakness and atrophy, cardiomyopathy and cardiac conduction defects, as well as insulin resistance have been identified to date: DM type 1 (DM1 or Steinert’s disease) and DM type 2 (DM2), which are caused by CTG trinucleotide expansion in 3′ untranslated region of *DMPK* (Dystrophia Myotonica Protein Kinase) gene and by CCTG tetranucleotide expansion in intron 1 of the *ZNF9/CNBP* (Zinc Finger 9/Cellular Nucleic acid Binding Protein) gene, respectively.

From the two types, DM1 is more common and severe. The severity of the disease is correlated with the degree of a tri-(CTG) nucleotide amplification. Molecular etiology of this disease is extremely complex because repeat expansion causes mis-regulation of molecular processes at different levels: in chromatin structure and local DNA methylation, DNA replication and stability, processing and translation of RNA, miRNA regulation, and proteostasis [113,114]. Due to the complexity of the molecular pathways affected, the cellular mechanisms in the development of pathology are poorly understood.

*DMPK* gene produces several alternatively spliced isoforms of serine/threonine kinase of the Rho kinase family. The most abundant isoform is mainly expressed in muscle [115]. In skeletal muscle, DMPK is localized predominantly in type 1 muscle fibers, neuromuscular junctions, and in cardiac muscle—in intercalated discs and Purkinje fibers. Among genes undergoing aberrant splicing due to mutated DMPK are muscle-specific genes and genes whose function is extremely important in striated muscle: of cardiac troponin T and fast skeletal muscle troponin T, muscle chloride channel ClC-1, insulin receptor, a large abundant protein of striated muscle titin, sarcomeric protein Cypher/ZASP, a calcium release channel in the sarcoplasmic reticulum RYR1 (ryanodine receptor 1) [114]. In addition to trans-dominant effects on distantly located genes, mRNA from expanded allele is inaccessible for translation, and this results in a decrease in the abundance of DMPK protein, which is also of pathological significance.

Functions of DMPK remain mostly unclear. Enzymatic substrates for this kinase in striated muscle cells include myogenin, the β-subunit of the L-type calcium channels, ion channel in plasma membrane phospholemman, and myosin phosphatase-target subunit 1 [116]. One of the DM1 roles presumably links it with laminopathies. DMPK is thought to be a structural component of the nuclear lamina. It is localized in the nuclear envelope, interacts with lamin A/C, and is considered necessary for maintaining the structure of the nuclear lamina [117].

Moreover, it was found that some DMPK isoforms are anchored on the cytosolic side of the sarcoplasmic reticulum and the outer mitochondrial membrane [118]. *DMPK* expression affects, among other things, the processes, in which mitochondria play a key role, such as maintaining Ca^2+^ homeostasis and apoptosis. The signs of *DMPK*-related mitochondrial dysfunction have been described, although its contribution to the DM1 pathogenesis is only beginning to be clarified. Swollen morphology and abnormalities in the ultrastructural organization of mitochondria have been described in muscle fibers from *DMPK^-/-^* mice [119]. In addition, the skeletal muscles of DM1 patients display abnormal mitochondrial accumulation in degenerated myofibrils [120]. Increased ROS levels and intensified apoptotic cell death were observed in DM1 patients’ muscle biopsies [121] and myotubes derived from DM1 primary myoblasts [122]. Reduced levels of Coenzyme Q10 (CoQ10), electron carrier in ETC, compared with normal controls, have been discovered in DM1 patients’ muscle samples in vitro and blood serum in vivo [123,124]. Notably, there was an inverse correlation between CoQ10 levels and the CTG expansion degree in DM patients.

Gramegna et al., using magnetic resonance spectroscopy, provided in vivo evidence for oxidative metabolism deficit in skeletal muscles of DM1 patients, and the impairment of oxidative metabolism was higher in more clinically affected patients [125].

Mitochondrial DMPK defends cells from oxidative stress [126]. However, being accumulated in the outer mitochondrial membrane, the largest of the DMPK-A isoforms causes undefined processes that lead to fragmentation and perinuclear clustering of mitochondria with abnormal ultrastructure, which threatens cell viability [127].

### 3.5. Barth Syndrome

Barth syndrome is a rare systemic disease involving cardiac and skeletal muscles. It was initially described as a disorder affecting only boys and characterized by dilated cardiomyopathy, skeletal muscle weakness, growth retardation, neutropenia, and sudden death due to the infections [128]. The genetic background of Barth syndrome was discovered in 1996 [129,130]. The causing mutations were spotted on the X-chromosome on gene *TAZ*. *TAZ* encodes the tafazzin protein, a phospholipid transacylase participating in cardiolipin remodeling via acylation of monolysocardiolipin [131].

Cardiolipin is a phospholipid located in the inner mitochondrial membrane, where it interacts with various proteins and provides stability to the cristae structure [132,133]. The presence of abnormal mitochondria found in biopsies samples from patients with Barth syndrome supported this genetic cause [134]. Analysis of mitochondria via electron microscopy showed great variability in mitochondrial size and volume, the presence of clusters of fragmented mitochondria inside nuclear invaginations, and the cristae density reduction [135,136].

Cardiolipin plays an important role in mitochondria health, participating in many critical processes—mitochondrial biogenesis, fusion and fission, cristae formation, respiration, protein import, mitophagy, and apoptosis—as has been demonstrated using animal and cellular models [132,137,138]. To date, around 160 *TAZ* mutations have been identified (https://www.barthsyndrome.org/research/tafazzindatabase.html (accessed on 15 June 2021). However, in carriers of the same mutations, Barth syndrome could be manifested by a wide range of phenotypic severity from asymptomatic carriers to neonatal death [139].

Thus, how exactly cardiolipin insufficiency modulates mitochondrial function and contributes to the pathogenesis of Barth syndrome is not yet fully understood and currently is under investigation. The first evidence of mitochondrial involvement was obtained using a yeast model with the deletion of the *TAZ1* gene. The authors demonstrated the destabilization of respiratory supercomplexes [140] and increased oxidative stress during respiratory growth at high temperature [141] in the absence of taffazin, as well as impaired mitophagy and reduced stability of mitochondrial membrane [142] in cells with genetically ablated cardiolipin [143]. Later, numerous animal models of Barth syndrome were developed. A recapitulated Drosophila model reduced locomotor activity and frequent mitochondrial abnormalities, mainly in the cristae membranes [144]. The role of tafazzin in heart development was assessed in a knock-down zebrafish model. A lack of tafazzin resulted in severe developmental and growth retardation and heart disorders similar to human heart failure. Aberrant cardiac development with a linear, nonlooped heart was accompanied by a hypomorphic tail, thus proving tafazzin’s role in the overall zebrafish development, especially of the heart [145].

The knowledge about tafazzin biology is largely based on data obtained from the mouse knock-down model. *TAZ* knock-down mice displayed pathological changes in mitochondria, myofibrils, and mitochondria-associated membranes in skeletal and cardiac muscles. Skeletal muscle mitochondria demonstrated disrupted cristae, cardiac mitochondria were significantly enlarged, and neighboring myofibrils were displaced. Analysis of cardiac function showed severe cardiac abnormalities, with left ventricular dilation, left ventricular mass reduction, and depression of fractional shortening and ejection fraction, while in skeletal muscle, a reduction in isometric contractile strength of the soleus was found [146,147]. Therefore, *TAZ* knock-down mouse model presented three principal pathological traits of Barth syndrome—cardiomyopathy, skeletal muscle wasting condition, and impaired mitochondria.

Further studies attempted to investigate the mechanisms underlying the mitochondrial dysfunction resulting from tafazzin deficiency in knock-down mice. Biochemical analysis of *TAZ* knock-down mice revealed a significant shift in bioenergetics and lipidomics in these animals [148]. Another study focused on the respiratory chain supercomplexes in cardiac tissue that were re-organized with the loss of complex II, resulting in decreased oxygen consumption rates and increased production of ROS [149]. Further analysis of hearts from *TAZ* knock-down mice revealed enhanced ROS production not accompanied by alteration in oxidative phosphorylation coupling efficiency or membrane potential. However, there was a reduction in mitochondrial coenzyme A levels and changes in fatty acid oxidation enzymes [150]. These mice displayed a decrease in glucose oxidation rates and an increase in fatty acid oxidation rates [151]. In contrast to these findings, a recent investigation showed that ROS levels were indistinguishable between *TAZ* knock-down mice and wild-type littermates. Thus, it can be concluded that cardiolipin deficiency in Barth syndrome pathogenesis is unlikely associated with boosted production of ROS [152].

The initial studies regarding cardiolipin role in calcium handling were performed using the yeast model and showed that cardiolipin was essential for providing stability and activity of mitochondrial calcium uniporter (MCU), the highly selective calcium channel complex in the inner mitochondrial membrane. The mutant yeast with tafazzin and cardiolipin genetic ablation had an enhanced turnover of MCU and impaired calcium uptake. The loss of functional cardiolipin in mammalian cell models and cardiac tissue from patients also resulted in reduced abundance and activity of MCU [153]. In the later studies, cardiomyocytes derived from human-induced pluripotent stem cells (iPSc) and cardiac-specific *TAZ* knock-out mice were used to analyze the effect of tafazzin depletion on Ca^2+^ handling. Tafazzin deficiency resulted in increased diastolic calcium and decreased calcium transient amplitude. Moreover, iPSc-derived cardiomyocytes from the patient with Barth syndrome had higher levels of mitochondrial ROS. The authors proposed ROS as a putative mechanistic link between *TAZ* mutations and calcium mishandling. ROS triggered calcium/calmodulin-dependent protein kinase II activation followed by RYR2 (ryanodine receptor2, a component of a calcium channel in the sarcoplasmic reticulum), phosphorylation, and an increase in calcium leak through RYR2 [154].

Due to its genetic aetiology, Barth syndrome is an attractive target for gene therapy. Recent advances in genetic engineering allowed the creation of a cardiac-specific *TAZ* knock-out mouse model [155]. Applied adeno-associated virus (AAV)-*TAZ* led to the expression of full-length tafazzin in the cardiac and skeletal muscles of knock-out mice, as measured by qPCR. The AAV gene therapy was strikingly efficient for neonatal survival rates, thus allowing to carry out the examinations of cardiac function in adult animals. Assessment of cardiomyopathic phenotype disclosed the significant decrease in cardiac hypertrophy, down-regulation of the genes critical for the heart failure program, decline of the heart fibrosis area, and reduction of apoptotic nuclei. The evidence from this study convincingly demonstrated the potency of gene therapy as an approach to rescue the cardiac and skeletal muscle function in Barth syndrome. However, the authors pointed out several considerations that should be taken into account when establishing the gene therapy product: evaluation of dose-response effect, evaluation of the ability of AAV to reverse the cardiac pathological phenotype, and evaluation of the persistence of the therapeutic response [155].

### 3.6. The Other Genes

As for another myopathy/cardiomyopathy-linked genes, the data about skeletal muscle bioenergetics are sporadic and scattered (Table S1). As with dystrophin, mutations that lead to loss or defects in the other components of dystrophin-glycoprotein complex and DGC-associated proteins also promote different forms of muscular dystrophy.

Within DGC, dystroglycans link dystrophin that associates with actin, to the extracellular matrix. Therefore, structural defects in the components of the dystroglycan subcomplex can result in the loss of linkage between the intracellular actin cytoskeleton and the extracellular matrix, disrupting structural support for sarcolemma. An extensively glycosylated protein α-dystroglycan binds laminin, the protein of basement membrane, via its carbohydrate residues. Transmembrane glycosyltransferase fukutin is involved in the glycosylation of α-dystroglycan in skeletal muscle. Mutations in the gene for fukutin, *FKTN*, and subsequent aberrant glycosylation of α-dystroglycan are responsible for dilated CM and several forms of congenital muscular dystrophy, such as LGMD2M. A recent study has shown that muscle-specific deletion of *FKTN* gene in mice resulted in 16% lower mitochondrial respiratory function and ~30% lower muscle strength compared with healthy littermate controls [156]. Besides, the expression of the gene for PGC1α, a primary transcription factor for mitochondrial biogenesis, was ~80% lower, which probably contributes to the development of mitochondrial defects.

Similarly, mutations in the genes of sarcoglycan subcomplex (encoding α, β, γ, and δ-sarcoglycan), which is integral to DGC, promote LGMD2C-2F indistinguishable from DMD by clinical manifestations. The expression profiling in human skeletal muscle from patients with LGMD2D, dystrophy caused by the ±-sarcoglycan deficiency, revealed a significantly reduced expression of many genes involved in mitochondrial function and energy metabolism [69]. The mitochondrial dysfunction was later confirmed in LGMD2D patients and a-sarcoglycan null mouse model: reduced mitochondrial content and activity, defective OxPhos accompanied by persistent impairment of mitochondrial biogenesis were shown. Along with these mitochondrial defects, authors detected the pathological epigenetic alterations on *PGC1α* promoter and showed that in a-sarcoglycan null muscle the *PGC1α* transcription and mitochondrial biogenesis could be restored using deacetylase inhibitor trichostatin A [157].

Prominent mitochondrial swelling was reported in skeletal muscle of *SGCD^-/-^* and *LAMA2^−/−^* mice lacking δ-sarcoglycan and laminin ±2-chain, respectively [77]. Laminin-2 (merosin) is the protein of basement membrane binding to DGC. Loss of its ±2 subunit encoded by the *LAMA2* gene causes merosin-deficient congenital muscular dystrophy (MDCA1), or congenital muscular dystrophy type 1A. Not so long ago, interesting results were obtained from simultaneous investigation of bioenergetic profiles of myogenic cells from MDCA1 and Leigh syndrome patients [158]. Leigh syndrome is a neurometabolic disease, which develops due to mutations in genes related to mitochondrial function, such as genes of ETC complexes’ proteins, resulting in mitochondrial energy generation defects. In both types of cells, the researchers discovered a dysregulated gene expression pattern of metabolism-related genes and concomitant bioenergetic impairment: reduced mitochondrial respiration and ATP production, increased glycolysis, and reduced fatty acid oxidation. The fact that structural defect (in this case, the defect of laminin) leads to functional alterations similar to one caused by a direct mitochondrial defect supports the concept of structural and mitochondrial interrelations in muscle cells. Apparently, in the highly structured intracellular configuration of the muscle, mitochondria are the functional integrators of the structural organization and metabolic/energetic systems of the cell.

To date, we know that the expression of some other mutated structural proteins of striated muscle cells, causing neuromuscular disorders that can be combined with cardiac diseases, also leads to mitochondrial/metabolic alterations (Table S1). These are, for example, sarcomeric protein β-myosin heavy chain (*MYH7*) [22,24]; the muscle-specific scaffolding protein of caveolae caveolin-3 (*CAV3*) [159,160,161]; and trans-sarcolemmal protein presumably involved in vesicle trafficking dysferlin (*DYSF*) [162,163,164].

Precisely how alterations induced by mutations in such different structural (though not only) proteins drive mitochondrial dysfunction in muscle cells remains unclear. The common element for all pathologies would appear to be an overall cellular response, which is a loss of cellular structural/functional integrity caused by disturbed cytoarchitectural infrastructure, ion homeostasis, and cell signaling. In this context, it is important to emphasize once again the fact that mitochondrial disturbances are observed earlier than structural damage to the muscle, as found in mouse models of muscle pathologies. In *mdx* mice, it has recently been established that disruption of mitochondrial appearance and function is one of the earliest pathological changes in dystrophic skeletal muscles, preceding the occurrence of muscle fiber abnormalities [65]. Mitochondrial impairment anticipates the onset of CM in model animals of DMD [165] and laminopathies [166]. Likewise, mitochondrial abnormalities are detected very early, before the appearance of obvious structural defects, both in cardiomyocytes and slow-twitch skeletal muscle, in desmin-null [89] and mutant desmin knock-in mice [167].

However, it is controversial whether the violation of bioenergetics is a cause or consequence in the pathogenesis of myopathy of any origin. In cardiac remodeling resulting from genetic aetiologies, mitochondria also display a spectrum of ultrastructural and functional defects that are believed to be implicated as a mechanism for energetic failure and oxidative stress. Such defects are well documented in dystrophinopathies and desminopathies [165,168]. Mitochondrial alterations share common features in cardiomyocytes and skeletal muscle cells, supporting the central involvement of these organelles in coupling between metabolism and contraction. However, as we described earlier using the example of *mdx* mice, pathogenic alterations of mitochondrial physiology may differ in cardiac and skeletal muscle. Skeletal and cardiac muscle mitochondria are different in some aspects, in particular, in Ca^2+^-responsiveness [169,170]. There are very few studies that would address simultaneously striated muscle metabolism in cardioskeletal myopathies; therefore, at the current stage of research, little is known about whether any bioenergetic changes are specific to skeletal muscle. Another open question is whether energetic/metabolic alterations in cardiac and skeletal muscle arise independently, by the same mechanism, due to the common primary structural defect, or resulting from mutations pathologic remodeling of myocardium in CM influence on skeletal muscle substrate metabolism and energetics.

Intriguing results, obtained in a mouse model of hypertrophic CM with cardiac-specific expression of human mutated *MYL2*, shed some light on skeletal muscle metabolic adaptation to cardiac pathologic conditions [171]. The product of the *MYL2* gene is the myosin regulatory light chain (RLC) which is expressed in both heart ventricles and slow-twitch skeletal muscles. Despite the fact that the expression of the R58Q-RLC mutant was restricted to the heart of mice, functional and structural changes (fiber-type remodeling with a decreased ratio of fiber type I/type II, lower contractile force) were discovered in slow-twitch soleus muscles, but not in fast-twitch muscles of transgenic mice. Besides, a quantitative proteomic approach revealed a common trend in the differential expression of cytoskeletal structural proteins, as well as proteins involved in metabolic processes and calcium handling in the hearts and soleus muscles of R58Q-RLC mutant mice, compared to wild type mice.

## 4. Conclusions

**Figure 1:**
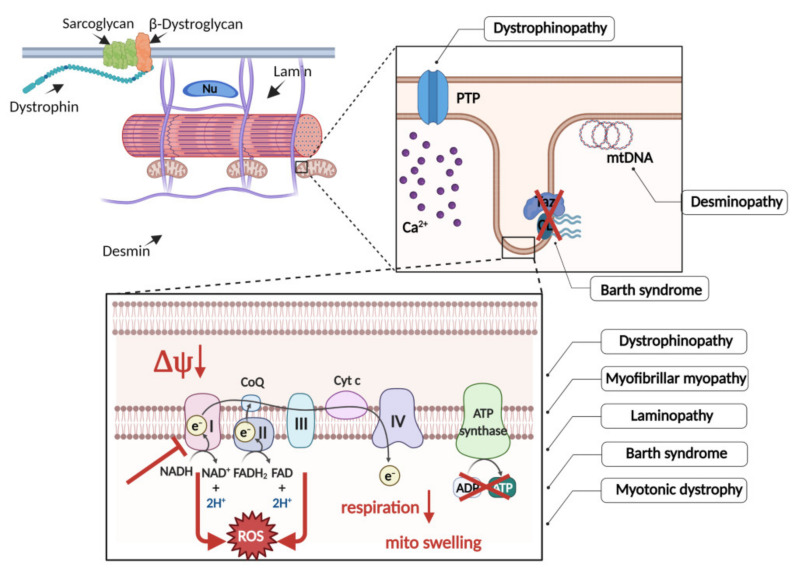
The mutations in different genes that lead to the disruption of the structural and functional integrity of muscle cell trigger the wide spectra of mitochondrial dysfunctions in specific skeletal muscle disorders.

Mounting evidence has shown that, regardless of the primary cause, mitochondrial dysfunction is a common feature of genetically determined skeletal muscle disorders associated with cardiomyopathies. Mitochondria are unique organelles that appear to serve as a sensory hub where various signals converge regarding the structural and functional integrity of the cell. Mitochondrial abnormalities occur in the early stage of the disease, and mitochondrial dysfunction is a simultaneous consequence and contributor to skeletal muscle pathologies. Violation of bioenergetics metabolism leads to secondary dysregulation of cellular processes critical for skeletal muscle functioning and further progression of pathology. The manifestations of dysfunction are similar in various disorders, as we summarize in the Figure 1. More works are also needed to find out whether there are metabolic/energetic changes specific for each mutation/pathology. Thus, regulation of the mitochondrial morphology, physiology, and pathological function might provide a basis to design therapeutic strategies to improve skeletal muscle metabolic health. The need is obvious to gain insight into molecular mechanisms underlying the mitochondrial dysfunction in striated muscles.

## Data Availability

Not applicable.

## Supplementary Materials

The following are available online at https://www.mdpi.com/article/10.3390/ijms22147349/s1, Table S1: Summary of mitochondrial and metabolic abnormalities in skeletal muscle in genetic neuromuscular disorders associated with cardiomyopathies.
