# Supplementary material for: Skeletal Muscle Mitochondria Dysfunction in Genetic Neuromuscular Disorders with Cardiac Phenotype
Source: Int J Mol Sci. 2021 Jul 8;22(14):7349. doi: 10.3390/ijms22147349 (PMC8307986; doi:10.3390/ijms22147349)
Supplement: Supplementary file 1 [file ijms-22-07349-s001.zip › ijms-1263082-supplementary.pdf]

Table S1

Summary of mitochondrial and metabolic abnormalities in skeletal muscle in genetic neuromuscular disorders associated with cardiomyopathies

Abbreviations for the table

ARVC, arrythmogenic right ventricular cardiomiopathy; COX, cytochrome C oxidase or complex IV; DCM, dilated cardiomyopathy; DMD, Duchenne muscular dystrophy; ETC, electron transport chain; FPLD, familial partial lipodystrophy; HCM, hypertrophic cardiomyopathy; LGMD, limb-girdle muscular dystrophy; LVNC, left ventricular noncompaction cardiomyopathy; mtDNA, mitochondrial DNA; MMP, mitochondrial membrane potential; MPTP, mitochondrial permeability transition pore; nNOS, neuronal NO synthase; OxPhos, oxidative phosphorylation; RCM, restrictive cardiomyopathy; ROS, reactive oxygen species; TCA, tricarboxylic acid cycle

Mutated gene/protein	Cardiac/Muscular phenotype	Model	Mitochondrial abnormalities					Metabolic defects
			Mitochondrial content, morphology and dynamics	Spatial distribution	Mitochondrial function	mtDNA	Mitochondrial proteome	
DYS/dystrophin	DCM, LVNC/ Duchenne/Becker muscular dystrophy	Patients' muscle			↓Up to 50% in the activity of all respiratory chain-linked enzymes<br>↓Maximal rates of respiration in permeabilized muscle fibres and isolated mitochondria [1]<br>↓Large scale down-regulation of nuclear encoded mitochondria gene expression [2]			Deranged metabolic gene expression [2]<br>Abnormal intrafibral lipid accumulation [3]<br>↓nNOS expression and enzyme activity [4], [5]<br>↓Enzymes of purine nucleotide cycle (activity and content) [6], [7]<br>Deregulation of lipid metabolism, ↓ fat oxidation [8], [9], for review [10]<br>↓Activity of glycolytic enzymes [11]
		Myogenic cells derived from DMD patients						↓mRNA encoding enzymes involved in mitochondrial beta-oxidation<br>↓Fatty acid transport into mitochondria, alterations in the lipid composition of muscle membranes [12]
		mdx mice muscle	Loss of mitochondrial biomass [13], [14]<br>Rounded swollen morphology and grossly disrupted internal structure	↓Subsarcolemmal mitochondria, redistribution of intermyofibrillar mitochondria [18]	↓Activity of ETC complexes and ETC-linked enzymes [1], [13], [16]<br>↑Susceptibility to Ca^{2+}-induced MPTP opening *in vitro* [13], [15], [19]<br>↓Mitochondrial respiration	↓mtDNA copy number [16]<br>↓Mitochondrial mRNA [24]	↑ UQCRC2, subunit of complex III [18]<br>Altered levels of proteins related to mitochondria (↑Drp1, MFN2,	↓nNOS expression and enzyme activity [4], [5]<br>↓Cytosolic enzymes of glycolysis [25], [26], TCA cycle [25]<br>Alterations in whole-

			[15]<br>↓Cristae number, aberrant structure, vacuolization [16]<br>Swelling, enlargement [17]		capacity in permeabilized muscle fibres [1], [20], [21] and isolated mitochondria [1], [17], [22]<br>↓Coupled respiration [20], [22]<br>↓Spare respiratory capacity [23]<br>↓Coupling of oxidative phosphorylation, maximal ATP synthesis capacity *in vivo* [18]		Parkin, ↓PARL) [16]<br>Altered levels of putative MPTP proteins:<br>↑ANT2<br>↓ANT1, cyclophilin D, ATP synthase, VDAC1 [19]<br>↑MCUb, the dominant negative subunit of Ca2+ uniporter (MCUb) [19]	tissue phospholipid composition (for review [10])
		Myoblasts derived from *mdx* mice	Disorganized mitochondrial network [27]		↓Oxygen consumption capacity<br>↑MMP<br>↑ROS formation [27]		↓Respiratory chain components and ATP synthase [27]	↑Glycolysis [27]
DES/desmin	HCM, DCM, RCM, LVNC, ARVC / Desmin-related myopathy	*DES*$^{-/-}$ mice	Rounded morphology with distorted inner membranes, enlargement [28]<br>Swollen/ disintegrating morphology [29]	Disorganized mitochondrial network, alterations in mitochondrial distribution [29], [30]<br>Abnormal clustering of subsarcolemmal clumps of mitochondria [31], [30], [28] and decrease in intermyofibrillar mitochondria [30]	↓Maximal respiration of slow oxidative skeletal muscle fibre mitochondria *in situ* [30]			

		Des knock-in mouse model expressing mutant desmin	Swelling and vacuolization [32]<br>Enlarged mitochondria [33]	Focal mitochondrial depletion/accumulation: the aggregation in the subsarcolemmal space and depletion in the intermyofibrillar space [33]	↑Mitochondrial Ca^{2+} level [32]<br>↓Citrate synthase activity [33]	↓mtDNA copy number, large-scale deletions [33]	↓ETC complexes [33]	
		Patients' muscle	↓Mitochondrial content [34], [35]<br>Enlarged mitochondria [33]<br>Paracrystalline inclusions [36]	Focal mitochondrial depletion/accumulation [37], [33], [35], [38], [39]<br>Focally increased number of mitochondria, often in clusters, with altered distribution [40]	↓Complex I activity in intact skeletal muscle fibres *in vivo* [37]<br>↓ Activity of respiratory chain enzymes in skeletal muscle homohenates, permeabilized muscle fibres, and isolated mitochondria [40]<br>↓Respiratory chain enzyme activity [34]	Clonally-expanded mtDNA deletions [35]<br>↓mtDNA content [34]<br>↓mtDNA copy number, large-scale deletions [33]	↓Content of mitochondrial respiratory chain components [35], [40]	
		Cell cultures *in vitro* expressing mutant desmin	↑Mitochondrial fragmentation [41]	↑Clustering of mitochondria in the perinuclear region [37]	↓MMP<br>↓Mitochondrial respiration<br>↑ADP/ATP ratio [41]<br>↓Calcium uptake [42]	↑mtDNA release [41]		
		Primary myoblasts from patient's skeletal					Differential expression of proteins associated with regulation of	

		muscles					MPTP [43]	
CRYAB/αB-crystalline, *LDB3*/ Cypher/ZASP, *FLNC*/filamin C, *MYOT*/ myotilin	HCM, DCM, LVNC/Myofibrillar myopathy	Patients' muscle	↓Mitochondrial mass [35]<br>Abnormal concentric or missing cristae, dense rectangular inclusions [44]	Mitochondrial displacement and redistribution: depletion of mitochondria in the centre and accumulation in the subsarcolemmal region of many fibres [35], [38]	↓Complex IV activity [44]<br>Nonspecific abnormalities of the oxidative/mitochondrial enzyme stainings [45]	Clonally-expanded mtDNA deletions [35]<br>Multiple mtDNA deletions in COX-deficient fibres [44]	Deficiency of complexes I and IV [35]	
LMNA/ lamin A/C	HCM, DCM, RCM, LVNC/ LGMD1B, Congenital muscular dystrophy, Emery-Dreifuss muscular dystrophy, Hutchinson-Gilford progeria syndrome, Familial partial lipodystrophy type 2 (FPLD2), Mandibuloacral dysplasia type A, Charcot-Marie-Tooth neuropathy	FPLD patients, *in vivo* and *in vitro* (cultured primary myotubes)			↓Complex I genes expression [46]			↓Glucose oxidation<br>↑Fatty acid oxidation, incomplete<br>↓Some glycolysis genes expression [46]
		Myogenic cell cultures *in vitro* expressing mutant lamin A			↑Proton leak<br>↓Coupling of mitochondrial respiration<br>Altered expression of OxPhos, TCA, and aerobic respiration pathways genes [47]			↓Glycolysis<br>↓Glycolysis genes expression [47]
CAV3/caveolin-3 (CAV3)	HCM, DCM/Distal myopathy with caveolin defect, Idiopathic hyperCK-emia,	CAV3 p.P104L knock-in mice, quadriceps muscles	Damaged, probably degenerating mitochondria [48]				Altered expression of mitochondrial proteins, involved in different	

	LGMD1C, Rippling muscle disease						mitochondrial processes (global proteomal profiling) [48]	
		CAV3-depleted and p.P104L expressing myogenic cell cultures *in vitro*	Fragmented/steroid morphology<br>↓Mitochondrial size [49]		↓Mitochondrial respiratory capacity<br>↓ATP/ADP ratio<br>↑Superoxide production<br>Depolarization of MMP [49]		↓Proteins implicated in mitochondrial biogenesis and function (MFN2, OPA, PGC1α) [49]	Disordered glucose metabolism (↓glucose uptake, ↓glycogen synthesis after insulin stimulation) [50]
		CAV3$^{-/-}$ mice, gastrocnemius muscle	↓Mitochondrial density [49]				Altered levels of proteins linked to mitochondrial function<br>↓SDHA, COX IV, PGC1a, ANT1, MnSOD<br>↑Drp1 [49]	
SGCA/α-sarcoglycan	DCM, ARVC/ LGMD2D	Patients' muscle	↓Mitochondrial content [51]		↓Complexes II and IV activity [51]<br>↓Expression of genes involved in mitochondrial function and energy metabolism [2]	↓mtDNA [51]		
		S*GCA*$^{-/-}$ mice	↓Mitochondrial density<br>Impairment of mitochondrial biogenesis [51]		↓Expression of genes encoding PGC1α and ETC subunits (cytochrome b, ATPase, COX subunit IV)<br>↓Oxidative metabolism in permeabilized muscle fibres [51]	↓mtDNA [51]	↓Levels of mitochondrial proteins (PGC1α, COX IV, VDAC-1, mtCO1) [51]	

SGCD/ δ - sarcoglycan	DCM, HCM/ LGMD2F	$SGCD^{-/-}$ mice	Swelling [52]					
LAMA2/ laminin-2 (merosin)	DCM/ Congenital muscular dystrophy type 1A (MDCA1)	$LAMA2^{-/-}$ mice						Altered expression of proteins involved in different metabolic processes (proteomal profiling [53]
		Primary myogenic cells from MDCA1 patients			↓Mitochondrial respiration<br>↓ATP production [54]			↑Glycolysis<br>↓Fatty acids oxidation<br>[54]
MYH7/ β-myosin heavy chain	HCM, DCM, LVNC/ Distal myopathy (Laing), Hyaline body myopathy, Myosin storage myopathy and cardiomyopathy	Patients' skeletal muscle	Focal patterns of crystolysis [55]	Focal reduction in mitochondria in the centre of type I oxidative fibres [56]			mtDNA mutations [55]	
		Patients' calf muscles *in vivo*, ^{31}P magnetic resonance spectroscopy			↓Oxidative capacity of the muscle [57]			
DMPK/ DMPK (dystrophia myotonica protein kinase)	(Rarely) HCM, DCM, LVNC/Myotonic dystrophy (Dystrophia Myotonica 1, DM1)	$DMPK^{-/-}$ mice	Swollen morphology, abnormal ultrastructural organization [58]					Impaired insulin signalling in muscle tissues<br>↓Glucose uptake [59]
		Patients' skeletal muscle		Accumulation of mitochondria between disorganized myofibrils [60]	↑Apoptotic cell death [61]			
		Myotubes derived from primary DM1			↑ROS level<br>↑Apoptotic cell death<br>[62]			

		myoblasts						
		Patients' skeletal muscle Blood serum			↓Coenzyme Q10 [63], [64]			
		Patients' calf muscles *in vivo*, ^{31}P magnetic resonance spectroscopy			Impairment of oxidative metabolism [65]			
ZNF9/*CNBP* / ZNF9/CNBP	DCM, LVNC/Myotonic dystrophy (Dystrophia Myotonica 2, DM2)	Myotubes derived from primary DM2 myoblasts, proteome analysis					Reduction in the profile of proteins involved in mitochondrial biogenesis and function [66]	↓Enzyme involved in fatty acids degradation [66]
DYSF/dysferlin	DCM/ LGMD2B, Miyoshi myopathy	Patients' skeletal muscle	Paracrystalline mitochondrial inclusions [67]	↑Subsarcolemmal mitochondria [67]	↓Complexes I, III and IV activity [68]	↑mtDNA copy number [68]	↓Protein levels of complexes I, III, IV subunits [68] Deficiency of complexes I and IV [69]	
		Dysferlin-deficient mice Patients' skeletal muscle						Lipid droplets, high fatty tissue replacement of myofibres [70]

FKTN/ fukutin	DCM/LGMD2M, Fukuyama congenital muscular dystrophy, Walker-Warburg syndrome	Mice with muscle-specific deletion of *FKTN* gene			↓Mitochondrial respiratory function<br>↓Expression of the gene for PGC1α [71]			

References

1. Kuznetsov, A. V.; Winkler, K.; Wiedemann, F.R.; Von Bossanyi, P.; Dietzmann, K.; Kunz, W.S. Impaired mitochondrial oxidative phosphorylation in skeletal muscle of the dystrophin-deficient mdx mouse. *Mol. Cell. Biochem.* **1998**, *183*, 87–96, doi:10.1023/A:1006868130002.
2. Chen, Y.W.; Zhao, P.; Borup, R.; Hoffman, E.P. Expression profiling in the muscular dystrophies: Identification of novel aspects of molecular pathophysiology. *J. Cell Biol.* **2000**, *151*, 1321–1336, doi:10.1083/jcb.151.6.1321.
3. Bonsett, C.A.; Rudman, A. “Oil globules” in Duchenne muscular dystrophy - History, demonstration, and metabolic significance. *Med. Hypotheses* **1994**, *43*, 327–338, doi:10.1016/0306-9877(94)90112-0.
4. Brenman, J.E.; Chao, D.S.; Xia, H.; Aldape, K.; Bredt, D.S. Nitric oxide synthase complexed with dystrophin and absent from skeletal muscle sarcolemma in Duchenne muscular dystrophy. *Cell* **1995**, *82*, 743–752, doi:10.1016/0092-8674(95)90471-9.
5. Chang, W.; Iannacconet, S.T.; Lau, K.S.; Masters, Bet.Sue S.; Mcabe, T.J.; Mcmillan, K.; Padre, R.C.; Spencer, M.J.; Tidball, J.G.; Stull, J.T.; et al. Neuronal nitric oxide synthase and dystrophin-deficient muscular dystrophy. *Proc. Natl. Acad. Sci.* **1996**, *93*, pp. 9142-9147, doi: 10.1073/pnas.93.17.9142.
6. van Bennekom, C.A.; Oerlemans, F.T.; Kulakowski, S.; De Bruyn, C.H. Enzymes of purine metabolism in muscle specimens from patients with Duchenne-type muscular dystrophy. *Adv. Exp. Med. Biol.* **1984**, *165 Pt B*, 447–450, doi:10.1007/978-1-4757-0390-0_85.
7. Camiña, F.; Novo-Rodriguez, M.I.; Rodriguez-Segade, S.; Castro-Gago, M. Purine and carnitine metabolism in muscle of patients with Duchenne muscular dystrophy. *Clin. Chim. Acta* **1995**, *243*, 151–164, doi:10.1016/0009-8981(95)06164-9.
8. Sharma, U.; Atri, S.; Sharma, M.C.; Sarkar, C.; Jagannathan, N.R. Skeletal muscle metabolism in Duchenne muscular dystrophy (DMD): An in-vitro proton NMR spectroscopy study. *Magn. Reson. Imaging* **2003**, *21*, 145–153, doi:10.1016/S0730-725X(02)00646-X.
9. Carroll, J.E.; Villadiego, A.; Brooke, M.H. Increased long chain acyl CoA in duchenne muscular dystrophy. *Neurology* **1983**, *33*, 1507–1510,

doi:10.1212/wnl.33.11.1507.

10. Saini-Chohan, H.K.; Mitchell, R.W.; Vaz, F.M.; Zelinski, T.; Hatch, G.M. Thematic review series: Genetics of human lipid diseases: Delineating the role of alterations in lipid metabolism to the pathogenesis of inherited skeletal and cardiac muscle disorders. *J. Lipid Res.* **2012**, *53*, 4–27, doi: 10.1194/jlr.R012120.

11. Chi, M.M.Y.; Hintz, C.S.; McKee, D.; Felder, S.; Grant, N.; Kaiser, K.K.; Lowry, O.H. Effect of Duchenne muscular dystrophy on enzymes of energy metabolism in individual muscle fibers. *Metabolism* **1987**, *36*, 761–767, doi:10.1016/0026-0495(87)90113-2, doi:10.1016/0026-0495(87)90113-2.

12. Le Borgne, F.; Guyot, S.; Logerot, M.; Beney, L.; Gervais, P.; Demarquoy, J. Exploration of Lipid Metabolism in Relation with Plasma Membrane Properties of Duchenne Muscular Dystrophy Cells: Influence of L-Carnitine. *PLoS One* **2012**, *7*, 49346, doi:10.1371/journal.pone.0049346.

13. Godin, R.; Daussin, F.; Matecki, S.; Li, T.; Petrof, B.J.; Burelle, Y. Peroxisome proliferator-activated receptor γ coactivator 1-α gene transfer restores mitochondrial biomass and improves mitochondrial calcium handling in post-necrotic mdx mouse skeletal muscle. *Authors. J. Physiol. C* **2012**, *590*, 5487–5502, doi:10.1113/jphysiol.2012.240390.

14. Jahnke, V.E.; Van Der Meulen, J.H.; Johnston, H.K.; Ghimbovschi, S.; Partridge, T.; Hoffman, E.P.; Nagaraju, K. Metabolic remodeling agents show beneficial effects in the dystrophin-deficient mdx mouse model. *Skelet. Muscle* **2012**, *2*, 16, doi:10.1186/2044-5040-2-16.

15. Pauly, M.; Daussin, F.; Burelle, Y.; Li, T.; Godin, R.; Fauconnier, J.; Koechlin-Ramonatxo, C.; Hugon, G.; Lacampagne, A.; Coisy-Quivy, M.; et al. AMPK activation stimulates autophagy and ameliorates muscular dystrophy in the mdx mouse diaphragm. *Am. J. Pathol.* **2012**, *181*, 583–592, doi:10.1016/j.ajpath.2012.04.004.

16. Moore, T.M.; Lin, A.J.; Strumwasser, A.R.; Cory, K.; Whitney, K.; Ho, T.; Ho, T.; Lee, J.L.; Rucker, D.H.; Nguyen, C.Q.; et al. Mitochondrial Dysfunction Is an Early Consequence of Partial or Complete Dystrophin Loss in mdx Mice. *Front. Physiol.* **2020**, *11*, 690, doi:10.3389/fphys.2020.00690.

17. Rybalka, E.; Timpani, C.A.; Cooke, M.B.; Williams, A.D.; Hayes, A. Defects in mitochondrial ATP synthesis in dystrophin-deficient Mdx skeletal muscles may be caused by complex I insufficiency. *PLoS One* **2014**, *9*, doi:10.1371/journal.pone.0115763.

18. Percival, J.M.; Siegel, M.P.; Knowels, G.; Marcinek, D.J. Defects in mitochondrial localization and ATP synthesis in the mdx mouse model of Duchenne muscular dystrophy are not alleviated by PDE5 inhibition. *Hum. Mol. Genet.* **2013**, *22*, 153-167, doi:10.1093/hmg/dds415. doi:10.1093/hmg/dds415.

19. Dubinin, M. V.; Talanov, E.Y.; Tenkov, K.S.; Starinets, V.S.; Mikheeva, I.B.; Sharapov, M.G.; Belosludtsev, K.N. Duchenne muscular dystrophy is associated with the inhibition of calcium uniport in mitochondria and an increased sensitivity of the organelles to the calcium-induced permeability transition. *Biochim. Biophys. Acta - Mol. Basis Dis.* **2020**, *1866*, doi:10.1016/j.bbadis.2020.165674.

20. Gaglianone, R.B.; Santos, A.T.; Bloise, F.F.; Ortiga-Carvalho, T.M.; Costa, M.L.; Quirico-Santos, T.; da Silva, W.S.; Mermelstein, C. Reduced mitochondrial respiration and increased calcium deposits in the EDL muscle, but not in soleus, from 12-week-old dystrophic mdx mice. *Sci. Rep.* **2019**, *9*, 1–10, doi:10.1038/s41598-019-38609-4.

21. Passaquin, A.C.; Renard, M.; Kay, L.; Challet, C.; Mokhtarian, A.; Wallimann, T.; Ruegg, U.T. Creatine supplementation reduces skeletal muscle degeneration and enhances mitochondrial function in mdx mice. *Neuromuscul. Disord.* **2002**, *12*, 174–182, doi:10.1016/S0960-8966(01)00273-5.

22. Liang, R.C.R. Studies on mitochondria from dystrophic skeletal muscle of mice. *Biochem. Med. Metab. Biol.* **1986**, *36*, 172–178, doi:10.1016/0885-4505(86)90121-0.

23. Schuh, R.A.; Jackson, K.C.; Khairallah, R.J.; Ward, C.W.; Spangenburg, E.E. Measuring mitochondrial respiration in intact single muscle fibers. *Am. J. Physiol. - Regul. Integr. Comp. Physiol.* **2012**, *302*, 712–719, doi:10.1152/ajpregu.00229.2011.

24. Gannoun-Zaki, L.; Fournier-Bidoz, S.; Le Cama, G.; Chambon, C.; Millasseaub, P.; Lkger, J.J.; Dechesnea, C.A. Down-regulation of mitochondrial mRNAs in the mdx mouse model for Duchenne muscular dystrophy; *FEBS Letters* **1995**, *375*, 268-272, doi: 10.1016/0014-5793(95)01225-4.

25. Chinet, A.E.; Even, P.C.; Decrouy, A. Dystrophin-dependent efficiency of metabolic pathways in mouse skeletal muscles. *Experientia* **1994**, *50*, 602–605, doi:10.1007/BF01921731.

26. Wehling-Henricks, M.; Oltmann, M.; Rinaldi, C.; Myung, K.H.; Tidball, J.G. Loss of positive allosteric interactions between neuronal nitric oxide synthase and phosphofructokinase contributes to defects in glycolysis and increased fatigability in muscular dystrophy. *Hum. Mol. Genet.* **2009**, *18*, 3439–3451, doi:10.1093/hmg/ddp288.

27. Onopiuk, M.; Brutkowski, W.; Wierzbicka, K.; Wojciechowska, S.; Szczepanowska, J.; Fronk, J.; Lochmüller, H.; Górecki, D.C.; Zabłocki, K. Mutation in dystrophin-encoding gene affects energy metabolism in mouse myoblasts. *Biochem. Biophys. Res. Commun.* **2009**, *386*, 463–466, doi:10.1016/j.bbrc.2009.06.053.

28. Kay, L.; Li, Z.; Mericskay, M.; Olivares, J.; Tranqui, L.; Fontaine, E.; Tiivel, T.; Sikk, P.; Kaambre, T.; Samuel, J.L.; et al. Study of regulation of mitochondrial respiration in vivo. An analysis of influence of ADP diffusion and possible role of cytoskeleton. *Biochim. Biophys. Acta - Bioenerg.* **1997**, *1322*, 41–59, doi:10.1016/S0005-2728(97)00071-6.

29. Milner, D.J.; Weitzer, G.; Tran, D.; Bradley, A.; Capetanaki, Y. Disruption of muscle architecture and myocardial degeneration in mice lacking desmin. *J. Cell Biol.* **1996**, *134*, 1255–1270, doi:10.1083/jcb.134.5.1255.

30. Milner, D.J.; Mavroidis, M.; Weisleder, N.; Capetanaki, Y. Desmin cytoskeleton linked to muscle mitochondrial distribution and respiratory function. *J. Cell Biol.* **2000**, *150*, 1283–1297, doi:10.1083/jcb.150.6.1283.

31. Li, Z.; Mericskay, M.; Agbulut, O.; Butler-Browne, G.; Carlsson, L.; Thornell, L.E.; Babinet, C.; Paulin, D. Desmin is essential for the tensile strength and integrity of myofibrils but not for myogenic commitment, differentiation, and fusion of skeletal muscle. *J. Cell Biol.* **1997**, *139*, 129–144, doi:10.1083/jcb.139.1.129.

32. Kostareva, A.; Sjöberg, G.; Bruton, J.; Zhang, S.J.; Balogh, J.; Gudkova, A.; Hedberg, B.; Edström, L.; Westerblad, H.; Sejersen, T. Mice expressing L345P

mutant desmin exhibit morphological and functional changes of skeletal and cardiac mitochondria. *J. Muscle Res. Cell Motil.* **2008**, *29*, 25–36, doi:10.1007/s10974-008-9139-8.

33. Winter, L.; Wittig, I.; Peeva, V.; Eggers, B.; Heidler, J.; Chevessier, F.; Kley, R.A.; Barkovits, K.; Strecker, V.; Berwanger, C.; et al. Mutant desmin substantially perturbs mitochondrial morphology, function and maintenance in skeletal muscle tissue. *Acta Neuropathol.* **2016**, *132*, 453–473, doi:10.1007/s00401-016-1592-7.

34. McCormick, E.M.; Kenyon, L.; Falk, M.J. Desmin common mutation is associated with multi-systemic disease manifestations and depletion of mitochondria and mitochondrial DNA. *Front. Genet.* **2015**, *6*, 199, doi:10.3389/fgene.2015.00199.

35. Vincent, A.E.; Grady, J.P.; Rocha, M.C.; Alston, C.L.; Rygiel, K.A.; Barresi, R.; Taylor, R.W.; Turnbull, D.M. Mitochondrial dysfunction in myofibrillar myopathy. *Neuromuscul. Disord.* **2016**, *26*, 691–701, doi:10.1016/j.nmd.2016.08.004.

36. Vernengo, L.; Chourbagi, O.; Panuncio, A.; Lilienbaum, A.; Batonnet-Pichon, S.; Bruston, F.; Rodrigues-Lima, F.; Mesa, R.; Pizzarossa, C.; Demay, L.; et al. Desmin myopathy with severe cardiomyopathy in a Uruguayan family due to a codon deletion in a new location within the desmin 1A rod domain. *Neuromuscul. Disord.* **2010**, *20*, 178–187, doi:10.1016/j.nmd.2010.01.001.

37. Schröder, R.; Goudeau, B.; Simon, M.C.; Fischer, D.; Eggermann, T.; Clemen, C.S.; Li, Z.; Reimann, J.; Xue, Z.; Rudnik-Schöneborn, S.; et al. On noxious desmin: Functional effects of a novel heterozygous desmin insertion mutation on the extrasarcomeric desmin cytoskeleton and mitochondria. *Hum. Mol. Genet.* **2003**, *12*, 657–669, doi:10.1093/hmg/ddg060.

38. Claeys, K.G.; Fardeau, M.; Schröder, R.; Suominen, T.; Tolksdorf, K.; Behin, A.; Dubourg, O.; Eymard, B.; Maisonobe, T.; Stojkovic, T.; et al. Electron microscopy in myofibrillar myopathies reveals clues to the mutated gene. *Neuromuscul. Disord.* **2008**, *18*, 656–666, doi:10.1016/j.nmd.2008.06.367.

39. Henderson, M.; De Waele, L.; Hudson, J.; Eagle, M.; Sewry, C.; Marsh, J.; Charlton, R.; He, L.; Blakely, E.L.; Horrocks, I.; et al. Recessive desmin-null muscular dystrophy with central nuclei and mitochondrial abnormalities. *Acta Neuropathol.* **2013**, *125*, 917–919, doi: 10.1007/s00401-013-1113-x.

40. Kubánek, M.; Schimerová, T.; Piherová, L.; Brodehl, A.; Krebsová, A.; Ratnavadivel, S.; Stanasiuk, C.; Hansíková, H.; Zeman, J.; Paleček, T.; et al. Desminopathy: Novel Desmin Variants, a New Cardiac Phenotype, and Further Evidence for Secondary Mitochondrial Dysfunction. *J. Clin. Med.* **2020**, *9*, 937, doi:10.3390/jcm9040937.

41. Smolina, N.; Khudiakov, A.; Knyazeva, A.; Zlotina, A.; Sukhareva, K.; Kondratov, K.; Gogvadze, V.; Zhivotovsky, B.; Sejersen, T.; Kostareva, A. Desmin mutations result in mitochondrial dysfunction regardless of their aggregation properties. *Biochim. Biophys. Acta - Mol. Basis Dis.* **2020**, *1866*, doi:10.1016/j.bbadis.2020.165745.

42. Smolina, N.; Bruton, J.; Sjoberg, G.; Kostareva, A.; Sejersen, T. Aggregate-prone desmin mutations impair mitochondrial calcium uptake in primary myotubes. *Cell Calcium* **2014**, *56*, 269–275, doi:10.1016/j.ceca.2014.08.001.

43. Chen, Y.; Zheng, J.; Chen, S.; Zhu, M.; Hong, D. Mitochondrial proteomics reveal potential targets involved in mitochondrial abnormalities of desminopathy.

Clin. Neuropathol. **2017**, *36*, 15–22, doi:10.5414/NP300969.

44. Joshi PR, Hauburger A, Kley R, Claeys KG, Schneider I, Kress W, Stoltenburg G, Weis J, Vorgerd M, Deschauer M, Hanisch F. Mitochondrial abnormalities in myofibrillar myopathies. *Clin Neuropathol*. **2014**, *33*, 134-42. doi: 10.5414/NP300693.

45. Reimann, J.; Kunz, W.S.; Vielhaber, S.; Kappes-Horn, K.; Schröder, R. Mitochondrial dysfunction in myofibrillar myopathy. *Neuropathol. Appl. Neurobiol.* **2003**, *29*, 45–51, doi:10.1046/j.1365-2990.2003.00428.x.

46. Boschmann, M.; Engeli, S.; Moro, C.; Luedtke, A.; Adams, F.; Gorzelniak, K.; Rahn, G.; Mähler, A.; Dobberstein, K.; Krüger, A.; et al. LMNA mutations, skeletal muscle lipid metabolism, and insulin resistance. *J. Clin. Endocrinol. Metab.* **2010**, *95*, 1634–1643, doi:10.1210/jc.2009-1293.

47. Ignatieva, E. V.; Ivanova, O.A.; Komarova, M.Y.; Khromova, N. V.; Polev, D.E.; Kostareva, A.A.; Sergushichev, A.; Dmitrieva, R.I. LMNA Mutations G232E and R482L Cause Dysregulation of Skeletal Muscle Differentiation, Bioenergetics, and Metabolic Gene Expression Profile. *Genes (Basel).* **2020**, *11*, 1057, doi:10.3390/genes11091057.

48. González Coraspe, J.A.; Weis, J.; Anderson, M.E.; Münchberg, U.; Lorenz, K.; Buchkremer, S.; Carr, S.; Zahedi, R.P.; Brauers, E.; Michels, H.; et al. Biochemical and pathological changes result from mutated Caveolin-3 in muscle. *Skelet. Muscle* **2018**, *8*, 28, doi:10.1186/s13395-018-0173-y.

49. Shah, D.S.; Nisr, R.B.; Stretton, C.; Krasteva-Christ, G.; Hundal, H.S. Caveolin-3 deficiency associated with the dystrophy P104L mutation impairs skeletal muscle mitochondrial form and function. *J. Cachexia. Sarcopenia Muscle* **2020**, *11*, 838–858, doi:10.1002/jcsm.12541.

50. Deng, Y.F.; Huang, Y.Y.; Lu, W.S.; Huang, Y.H.; Xian, J.; Wei, H.Q.; Huang, Q. The Caveolin-3 P104L mutation of LGMD-1C leads to disordered glucose metabolism in muscle cells. *Biochem. Biophys. Res. Commun.* **2017**, *486*, 218–223, doi:10.1016/j.bbrc.2017.02.072.

51. Pambianco, S.; Giovarelli, M.; Perrotta, C.; Latella, L.; Clementi, E.; De Palma, C. Reversal of Defective Mitochondrial Biogenesis in Limb-Girdle Muscular Dystrophy 2D by Independent Modulation of Histone and PGC-1α Acetylation. *Cell Reports* **2016**, *17*, 3010–3023, doi:10.1016/j.celrep.2016.11.044.

52. Millay, D.P.; Sargent, M.A.; Osinska, H.; Baines, C.P.; Barton, E.R.; Vuagniaux, G.; Sweeney, H.L.; Robbins, J.; Molkentin, J.D. Genetic and pharmacologic inhibition of mitochondrial-dependent necrosis attenuates muscular dystrophy. *Nat. Med.* **2008**, *14*, 442–447, doi:10.1038/nm1736.

53. De Oliveira, B.M.; Matsumura, C.Y.; Fontes-Oliveira, C.C.; Gawlik, K.I.; Acosta, H.; Wernhoff, P.; Durbeej, M. Quantitative proteomic analysis reveals metabolic alterations, calcium dysregulation, and increased expression of extracellular matrix proteins in Laminin α2 Chain-deficient muscle. *Mol. Cell. Proteomics* **2014**, *13*, 3001–3013, doi:10.1074/mcp.M113.032276.

54. Fontes-Oliveira, C.C.; Steinz, M.; Schneiderat, P.; Mulder, H.; Durbeej, M. Bioenergetic Impairment in Congenital Muscular Dystrophy Type 1A and Leigh Syndrome Muscle Cells. *Sci. Rep.* **2017**, *7*, doi:10.1038/srep45272.

55. Arbustini, E.; Fasani, R.; Morbini, P.; Diegoli, M.; Grasso, M.; Dal Bello, B.; Marangoni, E.; Banfi, P.; Banchieri, N.; Bellini, O.; et al. Coexistence of mitochondrial DNA and β myosin heavy chain mutations in hypertrophic cardiomyopathy with late congestive heart failure. *Heart* **1998**, *80*, 548–558,

doi:10.1136/hrt.80.6.548.

56. Fananapazir, L.; Dalakas, M.C.; Cyran, F.; Cohn, G.; Epstein, N.D. Missense mutations in the β-myosin heavy-chain gene cause central core disease in hypertrophic cardiomyopathy. *Proc. Natl. Acad. Sci. U. S. A.* **1993**, *90*, 3993–3997, doi:10.1073/pnas.90.9.3993.

57. Thompson, C.H.; Kemp, G.J.; Taylor, D.J.; Conway, M.; Rajagopalan, B.; O'Donoghue, A.; Styles, P.; McKenna, W.J.; Radda, G.K. Abnormal skeletal muscle bioenergetics in familial hypertrophic cardiomyopathy. *Heart* **1997**, *78*, 177–181, doi:10.1136/hrt.78.2.177.

58. Reddy, S.; Smith, D.B.J.; Rich, M.M.; Leferovich, J.M.; Reilly, P.; Davis, B.M.; Tran, K.; Rayburn, H.; Bronson, R.; Cros, D.; et al. Mice lacking the myotonic dystrophy protein kinase develop a late onset progressive myopathy. *Nat. Genet.* **1996**, *13*, 325–335, doi:10.1038/ng0796-325.

59. Llagostera, E.; Catalucci, D.; Marti, L.; Liesa, M.; Camps, M.; Ciaraldi, T.P.; Kondo, R.; Reddy, S.; Dillmann, W.H.; Palacin, M.; et al. Role of myotonic dystrophy protein kinase (DMPK) in glucose homeostasis and muscle insulin action. *PLoS One* **2007**, *2*, 1134, doi:10.1371/journal.pone.0001134.

60. Ueda, H.; Shimokawa, M.; Yamamoto, M.; Kameda, N.; Mizusawa, H.; Baba, T.; Terada, N.; Fujii, Y.; Ohno, S.; Ishiura, S.; et al. Decreased expression of myotonic dystrophy protein kinase and disorganization of sarcoplasmic reticulum in skeletal muscle of myotonic dystrophy. *J. Neurol. Sci.* **1999**, *162*, 38–50, doi:10.1016/S0022-510X(98)00290-1.

61. Yamada, H.; Nakagawa, M.; Higuchi, I.; Horikiri, T.; Osame, M. Detection of DNA fragmentation of myonuclei in myotonic dystrophy by double staining with anti-emerin antibody and by nick end-labeling. *J. Neurol. Sci.* **2000**, *173*, 97–102, doi:10.1016/S0022-510X(99)00306-8.

62. Loro, E.; Rinaldi, F.; Malena, A.; Masiero, E.; Novelli, G.; Angelini, C.; Romeo, V.; Sandri, M.; Botta, A.; Vergani, L. Normal myogenesis and increased apoptosis in myotonic dystrophy type-1 muscle cells. *Cell Death Differ.* **2010**, *17*, 1315–1324, doi:10.1038/cdd.2010.33.

63. Tedeschi, D.; Lombardi, V.; Mancuso, M.; Martelli, F.; Sighieri, C.; Rocchi, A.; Tovani, S.; Siciliano, G.; Murri, L. Potential involvement of ubiquinone in myotonic dystrophy pathophysiology: New diagnostic approaches for new rationale therapeutics. *Neurol. Sci.* **2000**, *21*, S979–S980, doi:10.1007/s100720070014.

64. Siciliano, G.; Mancuso, M.; Tedeschi, D.; Manca, M.L.; Renna, M.R.; Lombardi, V.; Rocchi, A.; Martelli, F.; Murri, L. Coenzyme Q10, exercise lactate and CTG trinucleotide expansion in myotonic dystrophy. Proceedings of the Brain Research Bulletin; *Brain Res Bull*, **2001**, *56*, 405–410, doi: 10.1016/S0361-9230(01)00653-0.

65. Gramegna, L.L.; Giannoccaro, M.P.; Manners, D.N.; Testa, C.; Zanigni, S.; Evangelisti, S.; Bianchini, C.; Oppi, F.; Poda, R.; Avoni, P.; et al. Mitochondrial dysfunction in myotonic dystrophy type 1. *Neuromuscul. Disord.* **2018**, *28*, 144–149, doi:10.1016/j.nmd.2017.10.007.

66. Rusconi, F.; Mancinelli, E.; Colombo, G.; Cardani, R.; Da Riva, L.; Bongarzone, I.; Meola, G.; Zippel, R. Proteome profile in Myotonic Dystrophy type 2 myotubes reveals dysfunction in protein processing and mitochondrial pathways. *Neurobiol. Dis.* **2010**, *38*, 273–280, doi:10.1016/j.nbd.2010.01.017.

67. Gayathri, N.; Alefia, R.; Nalini, A.; Yasha, T.C.; Anita, M.; Santosh, V.; Shankar, S.K. Dysferlinopathy: Spectrum of pathological changes in skeletal muscle

tissue. *Indian J. Pathol. Microbiol.* **2011**, *54*, 350–354, doi:10.4103/0377-4929.81636.

68. Liu, F.; Lou, J.; Zhao, D.; Li, W.; Zhao, Y.; Sun, X.; Yan, C. Dysferlinopathy: Mitochondrial abnormalities in human skeletal muscle. *Int. J. Neurosci.* **2016**, *126*, 499–509, doi:10.3109/00207454.2015.1034801.

69. Vincent, A.E.; Rosa, H.S.; Alston, C.L.; Grady, J.P.; Rygiel, K.A.; Rocha, M.C.; Barresi, R.; Taylor, R.W.; Turnbull, D.M. Dysferlin mutations and mitochondrial dysfunction. *Neuromuscul. Disord.* **2016**, *26*, 782–788, doi:10.1016/j.nmd.2016.08.008.

70. Grounds, M.D.; Terrill, J.R.; Radley-Crabb, H.G.; Robertson, T.; Papadimitriou, J.; Spuler, S.; Shavlakadze, T. Lipid accumulation in dysferlin-deficient muscles. *Am. J. Pathol.* **2014**, *184*, 1668–1676, doi:10.1016/j.ajpath.2014.02.005.

71. Southern, W.M.; Nichenko, A.S.; Qualls, A.E.; Portman, K.; Gidon, A.; Beedle, A.M.; Call, J.A. Mitochondrial dysfunction in skeletal muscle of fukutin-deficient mice is resistant to exercise- and 5-aminoimidazole-4-carboxamide ribonucleotide-induced rescue. *Exp. Physiol.* **2020**, *105*, 1767–1777, doi:10.1113/EP088812.
